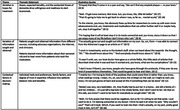# “I’m worth saving”‐ a qualitative study of people with dementia considering treatment with lecanemab

**DOI:** 10.1002/alz.094969

**Published:** 2025-01-09

**Authors:** Ayush Thacker, Anna L Parks, Daniel Dohan, Liliana A Ramirez Gomez, Christine S Ritchie, Joanna Paladino, Sachin J Shah

**Affiliations:** ^1^ Massachusetts General Hospital, Boston, MA USA; ^2^ University of Utah, Salt Lake City, UT USA; ^3^ University of California, San Francisco, San Francisco, CA USA; ^4^ Massachusetts General Hospital, Harvard Medical School, Boston, MA USA

## Abstract

**Background:**

As lecanemab becomes available to people living with dementia, there is a pressing need to understand how they weigh the potential benefits against costs. This study investigates how older adults perceive lecanemab’s risks and benefits and their approach to treatment decisions.

**Methods:**

Semi‐structured interviews of older adults undergoing evaluation in Neurology clinics for lecanemab eligibility at two academic medical centers. An interdisciplinary research team used rapid thematic analysis guided by the Ottawa Decision Support Framework.

**Results:**

22 people completed interviews (mean age 70 years, 36% women, 100% white). Preliminary themes included: 1) *Hopes, expected benefits, and the existential threat of dementia driving willingness and readiness to start lecanemab*. Hopes included more time with family and more time feeling like themselves by stalling the progression of cognitive decline and amyloid build‐up. Some expected benefits included “getting back to where I was” and “getting better, not worse.” Some patients pursued Lecanemab because it would be doing ‘something’ (versus nothing), given the fear and stress of dementia. 2) *Patients sought and obtained information* from different sources, including advocacy organizations, the Internet, and clinicians. Patients desired more information about their personal risk and wanted to hear more from patients who took the medication. 3) *Individual traits and preferences, family factors, and degree of trust in expertise influence how patients balance risks and benefits*. Some patients were willing to accept treatment at any cost, either due to the perceived inevitability of decline without intervention or because they tend to “look past the negatives” when making decisions. Others weighed risks (e.g., brain bleeding) and financial and logistical costs carefully, but supportive families, insurance coverage, and trust in the system helped in their decision to start treatment. A small proportion of people would not get the treatment as the costs outweighed their personal benefit.

**Conclusions:**

This group of people with mild dementia who are at the forefront of lecanemab treatment showed variation in hopes for treatment, information sought and obtained, and contextual factors in decision‐making, supporting the need for an individualized approach. These insights can guide future interventions to improve individualized decision‐making for lecanemab treatment.